# Later-Line Treatment with Lorlatinib in *ALK*- and *ROS1*-Rearrangement-Positive NSCLC: A Retrospective, Multicenter Analysis

**DOI:** 10.3390/ph13110371

**Published:** 2020-11-07

**Authors:** Maximilian J. Hochmair, Hannah Fabikan, Oliver Illini, Christoph Weinlinger, Ulrike Setinek, Dagmar Krenbek, Helmut Prosch, Markus Rauter, Michael Schumacher, Ewald Wöll, Romana Wass, Elmar Brehm, Gudrun Absenger, Tatjana Bundalo, Peter Errhalt, Matthias Urban, Arschang Valipour

**Affiliations:** 1 Department of Respiratory and Critical Care Medicine, Karl Landsteiner Institute of Lung Research and Pulmonary Oncology, Klinik Floridsdorf, Brünner Strasse 68, 1210 Vienna, Austria; hannah.fabikan@extern.gesundheitsverbund.at (H.F.); oliver.illini@gesundheitsverbund.at (O.I.); christoph.weinlinger@extern.gesundheitsverbund.at (C.W.); arschang.valipour@gesundheitsverbund.at (A.V.); 2 Institute of Pathology and Clinical Microbiology, Wilhelminenspital, Montleartstrasse 37, 1160 Vienna, Austria; ulrike.setinek@wienkav.at (U.S.); dagmar.krenbek@gesundheitsverbund.at (D.K.); 3 Department of Pathology and Bacteriology, Klinik Floridsdorf, Brünner Strasse 68, 1210 Vienna, Austria; 4 Department of Biomedical Imaging and Image-Guided Therapy, Medical University of Vienna, Währingergürtel 18–22, 1090 Vienna, Austria; helmut.prosch@meduniwien.ac.at; 5 Clinic of Pneumology, Klinikum Klagenfurt am Wörthersee, Feschnigstrasse 11, 9020 Klagenfurt am Wörthersee, Austria; markus.rauter@kabeg.at; 6 Ordensklinikum Linz Elisabethinen, Fadingerstrasse 1, 4020 Linz, Austria; michael.schumacher@ordensklinikum.at; 7 St. Vinzenz Krankenhaus Betriebs GmbH, Klostergasse 10, 6511 Zams, Austria; ewald.woell@krankenhaus-zams.at; 8 Department of Pneumology, Johannes Kepler University Linz, Krankenhausstrasse 26–30/Med Campus IV, 4020 Linz, Austria; elmar.brehm@kepleruniklinikum.at (E.B.); romana@wass.at (R.W.); 9 Department of Pneumology, Paracelsus Medical University, SALK, Müllner Hauptstrasse 46, 5020 Salzburg, Austria; 10 Department of Oncology, Medical University of Graz, Auenbruggerplatz 15, 8036 Graz, Austria; gudrun.absenger@medunigraz.at; 11 Landesklinikum Hochegg, Hocheggerstrasse 88, 2840 Hochegg, Austria; tatjana.bundalo@hochegg.lknoe.at; 12 Clinical Department of Pneumology, University Hospital Krems, Mitterweg 10, 3500 Krems, Austria; peter.errhalt@krems.lknoe.at; 13 Department of Respiratory and Critical Care Medicine, Klinik Floridsdorf, Brünner Strasse 68, 1210 Vienna, Austria; mathias.urban@gesundheitsverbund.at

**Keywords:** *ALK*-positive disease, *ROS1*-positive disease, ALK tyrosine kinase inhibitor treatment, sequential treatment, lorlatinib

## Abstract

In clinical practice, patients with *anaplastic lymphoma kinase (ALK)*-rearrangement–positive non–small-cell lung cancer commonly receive sequential treatment with ALK tyrosine kinase inhibitors. The third-generation agent lorlatinib has been shown to inhibit a wide range of *ALK* resistance mutations and thus offers potential benefit in later lines, although real-world data are lacking. This multicenter study retrospectively investigated later-line, real-world use of lorlatinib in patients with advanced *ALK*- or *ROS1*-positive lung cancer. Fifty-one patients registered in a compassionate use program in Austria, who received second- or later-line lorlatinib between January 2016 and May 2020, were included in this retrospective real-world data analysis. Median follow-up was 25.3 months. Median time of lorlatinib treatment was 4.4 months for *ALK*-positive and 12.2 months for *ROS*-positive patients. *ALK*-positive patients showed a response rate of 43.2%, while 85.7% percent of the *ROS1*-positive patients were considered responders. Median overall survival from lorlatinib initiation was 10.2 and 20.0 months for the *ALK*- and *ROS1*-positive groups, respectively. In the *ALK*-positive group, lorlatinib proved efficacy after both brigatinib and alectinib. Lorlatinib treatment was well tolerated. Later-line lorlatinib treatment can induce sustained responses in patients with advanced *ALK*- and *ROS1*-positive lung cancer.

## 1. Introduction

The *anaplastic lymphoma kinase (ALK)* fusion gene is a rare driver aberration that occurs only in 2% to 7% of all non-small cell lung cancer (NSCLC) cases, with a predilection in never- or light-smokers and in patients with adenocarcinoma histology [1,2,3,4,5,6]. Tyrosine kinase inhibitors (TKIs) targeting the *ALK* fusion gene have become the standard of care for these patients. Five ALK inhibitors including crizotinib, ceritinib, alectinib, brigatinib and lorlatinib have been approved to date for the treatment of *ALK*-positive lung cancer in the European Union and the United States [7,8,9,10,11,12].

Gainor et al. and Iwama et al. demonstrated the appearance of resistance mechanisms during prolonged treatment with TKIs. They differ between first-generation and next-generation ALK TKIs [13,14]. In patients who demonstrated treatment failure on the first-generation agent crizotinib, the main mechanisms of resistance include the expression of other oncogenes, changes in the copy number of the *ALK* gene, and activation of alternative pathways such as epidermal growth factor receptor (EGFR) or KIT signaling, while secondary *ALK* rearrangements were only found in 20% to 30% of patients. In contrast, *ALK* resistance mutations represented the major resistance mechanism (50% to 70%) in patients treated with ceritinib, alectinib, or brigatinib. This is thought to reflect the greater potency and selectivity of the next-generation TKIs compared to crizotinib.

Lorlatinib (PF-06463922) is a third-generation inhibitor of ALK and ROS1 tyrosine kinases that was developed to overcome resistance of earlier ALK inhibitors [15]. It is suitable for the treatment of patients with acquired resistance mutations such as ALK G1202R and ROS1 G2032R and, additionally, crosses the blood brain barrier better than earlier ALK-directed tyrosine kinase inhibitors (TKI) [9,16].

Lorlatinib is a macrocyclic compund with novel chemical scaffold to enhance penetration and prevent efflux in cells overexpressing P-glycoprotein and breast cancer resistant protein (BCRP). Construction of this macrocycle-based inhibitor has been driven by lipophilicity optimization to provide desirable pharmacological properties with high clinical potency and, compared to acyclic lactam analogues, metabolic stability, stereochemical specificity, improved cellular potency (119-fold) and ideal lipophilicity was obtained.

Moreover, Lorlatinib was potent against all ALK mutants clinically found and showed a 40825-fold increment in potency compared to crizotinib. Macrocycle-like conformations are often detecable for non-macrocycle small molecule ligands bound to their protein targets [17].

Consequently, macrocyclization as a drug development strategy has led to the generation of LOXO-195 for tropomyosin receptor kinase (TRK) fusion-positive solid tumors and the novel and highly efficient EGFR TKI BI-4020 [18]. In summary, Lorlatinib represents a new generation of TKIs exploiting the arrangement of established pharmacophores in macrocycles resulting in novel and highly potent therapeutics.

Sequential treatment furthermore raises the question of rebiopsy in patients with progressive *ALK*-positive lung cancer. Current knowledge regarding mutation patterns rests mostly on patient-derived cell line models [13], while clinical experience is limited. The efficacy of later-line ALK-targeted treatment does not necessarily depend on the mutation status, as the absence of *ALK* resistance mutations has not been shown to impact response rates or progression-free survival in crizotinib-resistant patients treated with second-generation ALK inhibitors [19,20,21]. Obtaining tissue can be difficult or even impossible in the advanced setting, because *ALK*-positive tumors tend to metastasize to the CNS. Liquid biopsy, which is well established in *EGFR*-mutant disease, is presently under validation for *ALK*-positive NSCLC [22]. The National Comprehensive Cancer Network NCCN [23] and International Association for the Study of Lung Cancer/College of American Pathologists/ Association for Molecular Pathology (IASCL/CAP/AMP) guidelines [24] do not recommend *ALK* mutation testing, while the European Society for Medical Oncology (ESMO) guidelines [25] recommend testing but add that this is not mandatory for treatment decisions.

Lorlatinib is a potent, CNS-active, highly selective ALK/ROS1 inhibitor that has been approved in the European Union, the US, Canada and Japan as a monotherapy for the treatment of adult patients with *ALK*-positive advanced NSCLC, whose disease has progressed after alectinib or ceritinib as the first ALK TKI, or crizotinib and at least one other ALK TKI [26,27]. In the pivotal phase II trial, lorlatinib has shown substantial overall and intracranial activity [28]. Real-world data on the use of this agent are lacking, however. Therefore, we retrospectively assessed the efficacy and tolerability of lorlatinib in patients with *ALK*-positive or *ROS1*-positive lung cancer.

## 2. Results

### 2.1. Patient Characteristics

This analysis included 51 patients with NSCLC, 48 (94.1%) with adenocarcinoma, 2 (3.9%) with adeno-squamous histology and 1 (1.9%) with squamous-cell carcinoma. An *ALK* rearrangement was harbored by 37 (72.5%) patients while 14 (27.5%) were *ROS1*-positive. All of them were of Caucasian ethnicity. At initial diagnosis, out of the 51 NSCLC patients, 2 (3.9%) patients presented stage I, while 6 (11.8%) and 43 (84.3%) had stage III and IV disease. Brain metastases were present in 28 patients, with an unknown CNS status in 4 patients. Overall characteristics were consistent with this kind of study population (Table 1).

The *ALK*-positive cohort consisted of 24 (64.9%) female and 13 (35.1%) male patients with a mean age at metastatic diagnosis of 53.0 years (range, 29–77). This subgroup included 3 (8.1%) current, 11 (29.7%) former and 23 (62.2%) never-smokers. Of the *ROS1*-positive group, seven patients were female and male (50.0%, 50.0%), at a mean age at metastatic diagnosis of 55.4 years (range, 26–82). Of those patients one (7.1%) was a current, five (35.7%) were former and eight (57.1%) never-smokers.

In the *ALK*-positive subgroup (n = 37), patients received multiple lines of therapy before switching to lorlatinib (range, 2–9). Prior lines of treatment consisted of ALK TKIs including crizotinib (n = 25, 67.6%), alectinib (n = 14, 37.8%), brigatinib (n = 27, 73.0%) and ceritinib (n = 21, 56.8%) or chemotherapy (n = 10, 27.0%). Before lorlatinib was administered, 10 (27.0%) patients already had received 1 line of TKI, 13 (35.1%) had 2 lines, 13 (35.1%) had 3 lines and 1 (2.7%) had 4 lines. On the other hand, *ROS1* rearranged patients (n = 14) mostly had two lines of prior therapy (range, 2–6). Their treatment consisted of crizotinib (n = 14, 100%), ceritinib (n = 3, 21.4%) or chemotherapy (n = 8, 57.1%). Eleven patients (78.6%) received one prior line of TKI and only three (21.4%) had two lines.

### 2.2. Efficacy

#### 2.2.1. ALK-Positive Patients

The median follow-up of the *ALK*-positive patients was 24.8 months. At the time of analysis, 11 (29.7%) out of the 37 *ALK*-positive patients were still on lorlatinib treatment.

**Figure 1:**
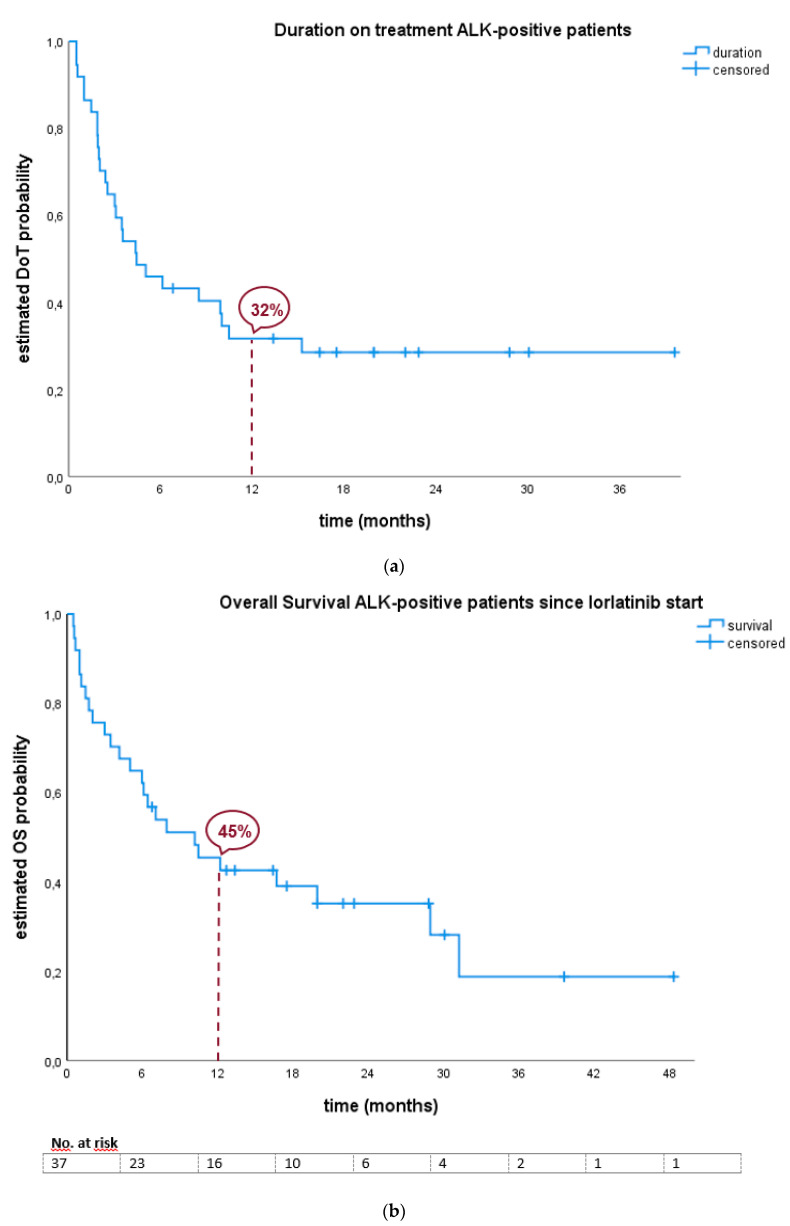

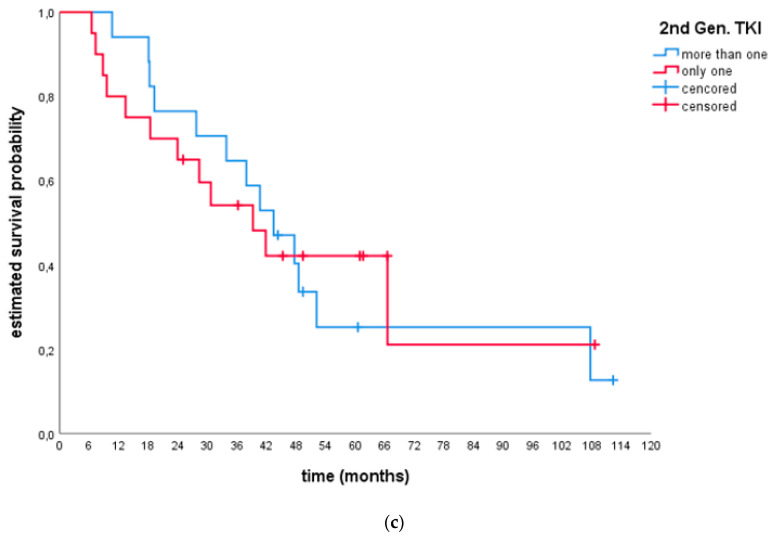
(**a**) Duration on treatment of *ALK* positive patients. (**b**) Overall Survival (OS) of *ALK* positive patients since start of lorlatinib treatment. (**c**) Comparison of OS since date of locally advanced or metastatic diagnosis between the two subgroups (only one prior second generation TKI or more).

Our patients showed a median duration on treatment (DoT) of 4.4 months (95% CI: 1.3; 7.6). (Table 2, Figure 1) Among the subgroups, the patients who received one line of prior ALK-TKI had a DoT of 4.4 months (n = 10; 95% CI: 0.5; 8.2) compared to 3.0 months (n = 14; 95% CI: 1.8; 4.2) in the group that received ≥ 3 prior lines of TKI with no significant difference between the subgroups. The subgroup with two prior lines of TKI has not yet reached a discontinuation rate of 50%.

Out of 37 *ALK*-positive patients, 12 (32.4%) were still alive on the date of analysis. Median overall survival (OS) from start of lorlatinib treatment was 10.2 months (95% CI: 3.6; 16.8) for all *ALK*-positive patients. Measured from the date of locally advanced or metastatic diagnosis, the median OS was 41.8 months (95% CI: 34.1; 49.5) (Table 2, Figure 1).

The 6-month, 1-year, 2-year and 5-year survival rates since date of advanced or metastatic diagnosis were estimated to be 100%, 86.5%, 70.3% and 33.3%. (Table 2) All patients were evaluated for response rates. The overall response rate (ORR) for *ALK*-positive patients was 43.2% (95% CI: 27.1; 60.5). Complete response (CR) and partial response (PR) occurred in 2.7% and 40.5% of these patients and the disease control rate (DCR) was 56.8% (95% CI: 39.5; 72.9) (Table 2).

The ORR for patients who received only one prior TKI was 40.0% (95% CI: 12.2; 73.8) compared to those with two prior lines of TKI who had an ORR of 53.8% (95% CI: 25.1; 80.8), on the contrary to the patients who received three or more lines of TKI. Those had an ORR of 35.7% (95% CI: 12.8; 64.9). No significant difference was observed (Table 2).

Eight patients had confirmed brain metastasis at initiation of lorlatinib and no following cranial radiotherapy. Out of these patients suitable for providing data for intracranial response, five (62.5%) showed an intracranial PR (1 stable disease (SD), 2 progressive disease (PD)).

- Efficacy after only 1 s generation TKI and ≥2 s generation TKIs:

Further subgroup analysis also showed no difference comparing the patients prior lines differing between patients who only received one second generation TKI since both had a median DoT of 4.4 months (n = 20; 95% CI: 1.1; 7.7) (n = 17; 95% CI: 0.2; 8.6) (Table 2, Figure 1).

Patients who received only one second generation TKI prior to lorlatinib showed a comparable survival time since date of locally advanced or metastatic diagnosis, with 39.2 months (95% CI: 21.7; 56.7) compared to 43.4 months (95% CI: 30.9; 55.9) in patients who received more than one second generation TKI. There was, significantly, no difference in survival (*p* = 0.003) between these groups (Table 2).

Those who received only one second generation TKI had a higher ORR of 50.0% (95% CI: 27.2; 72.8) compared to the subgroup with ≥2 prior second generation TKI with an ORR of 35.3% (95% CI: 14.2; 61.7).

- Efficacy after alectinib and brigatinib:

The ORR of the 19 patients who received brigatinib as their last line of therapy was 32.6% (95% CI: 12.6; 56.6), in contrast to patients who received alectinib previous to lorlatinib, with an ORR of 60% (n = 15; 95% CI: 32.3; 83.7). The median DoT in the alectinib group was 8.5 months (95% CI: 0.0; 17.2), which was higher compared to the brigatinib group with 3.5 months (95% CI: 1.8; 5.4).

Patients who received alectinib as their only second generation TKI showed a higher DoT of 10.0 months (n = 9; 95% CI: 0.0; 24.5) compared to those who received only brigatinib with 3.5 months (n = 11; 95% CI: 0.9; 6.1). The ORR of this alectinib group was 66.7% (95% CI: 29.9%; 92.5%) and therefore also higher than 36.4% (95% CI: 10.9%; 69.2%) in the patients who received only brigatinib. No difference in OS2 was observed with 39.2 months (95% CI: 26.3; 52.2) and 41.8 months (95% CI 7.2; 67.5) for the alectinib and the brigatinib group, respectively.

#### 2.2.2. ROS1-Positive Patients

Out of the 14 *ROS1*-positive patients, 6 patients (42.9%) were still on treatment and a median follow-up time of 32.2 months was reached.

**Figure 2:**
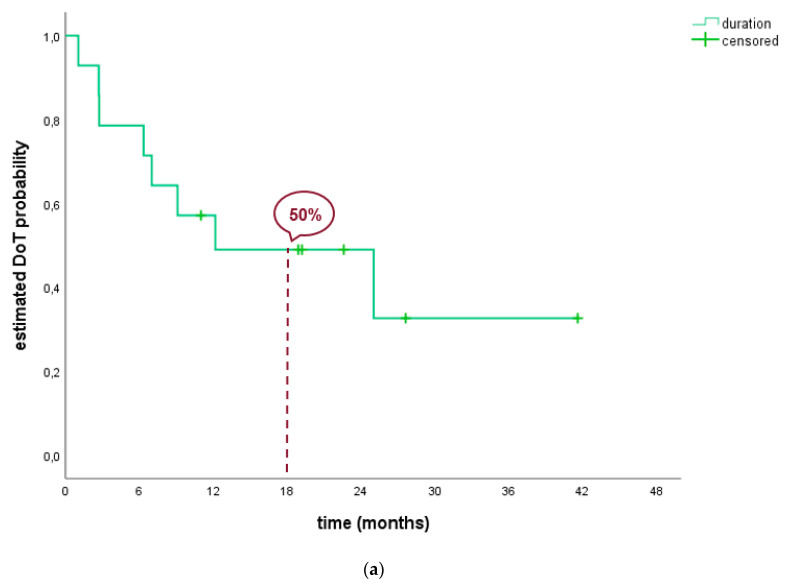

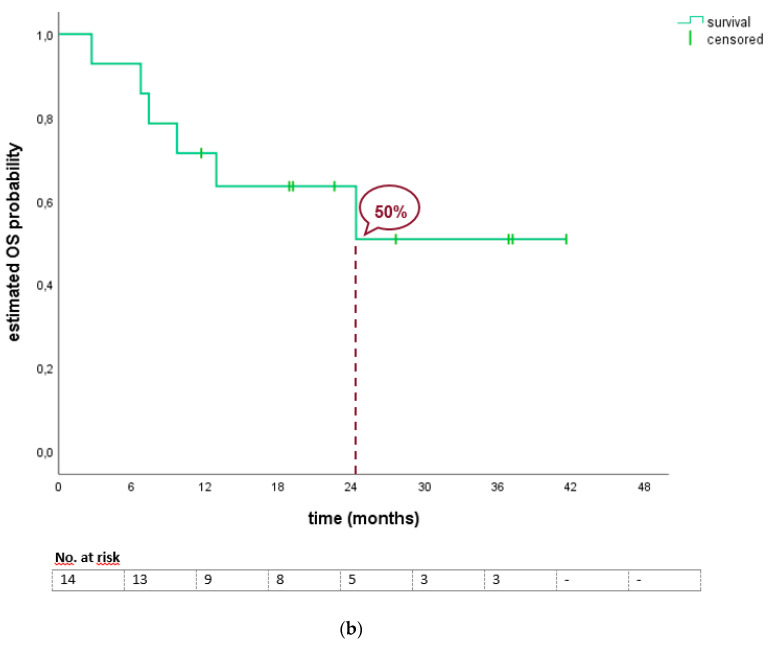
(**a**) Duration on treatment of *ROS1* positive patients. (**b**) Overall Survival of *ROS1* positive patients since start of lorlatinib treatment.

The median DoT of all patients was 12.2 months (95% CI: 0.0; 29.5) with a range of 1.0 to 41.6 months of treatment. Due to the lower number of patients and the homogeneity regarding the prior therapies, no subgroup analysis was conducted (Table 2, Figure 2).

On the date of analysis, 8 (57.1%) out of the 14 patients were still alive. The median OS was 20.0 months (95% CI: not reached (NR), range: 2.7; 41.6) since the start of lorlatinib and 40.0 months (95% CI: NR, range: 19.20–112.3 months) since the date of diagnosis of locally advanced or metastatic disease (Table 2, Figure 2).

The ORR was 85.7% (95% CI: 57.2; 98.2) with two (14.3%) patients having a complete response. The DCR was 92.9% (95% CI: 66.1; 99.8). Only one (7.1%) patient had a PD (Table 2).

### 2.3. Tolerability

Forty-nine patients received lorlatinib at the approved starting dose of 100 mg/day. Due to fears of toxicities, two patients were given 50 mg/day for one week before increasing to the standard dose. In both cases, the full dose was well tolerated. Dose reductions due to adverse events were necessary for 9 of the 51 patients (18%), with lorlatinib 25 mg/day as the lowest daily dose used.

Out of 51 patients, adverse evnts (AEs) occurred for 25 (49%). As presented in Table 3, the most common related adverse event was hyperlipidemia, affecting all of the 25 patients experiencing side effects, with grade ≥3 AEs seen in 9 patients (18%). Further differentiation shows hypercholesterolemia in 10 patients who experienced grade 1 or 2 events (20%), and 4 developing grade ≥3 events (8%). Grade 1/2 and grade 3/4 hypertriglyceridemia occurred for six (12%) and five (10%) patients, respectively.

Peripheral edema was observed in seven cases (14%), with six patients (12%) experiencing grade 1/2 events and one reported grade 3 event, necessitating a dose modification for two patients. Further rare grade 1 or 2 events included cognitive effects (6%), diarrhea (6%), bloating (2%), muscle weakness (2%) and myalgia (2%).

Rare grade ≥3 AEs included arthralgia (2%), fatigue (2%), pneumonitis (2%) and thrombosis (2%). In those patients experiencing grade 3/4 AEs, lorlatinib treatment was halted temporarily, and continued later at a reduced dose. One patient had to permanently discontinue due to toxicities.

## 3. Discussion

The optimal treatment sequence in the setting of *ALK*-positive NSCLC is of substantial clinical relevance. ALK TKIs are often particularly effective in these patients, thus changing the course of their disease, and their optimal sequence might affect patient outcomes to a considerable extent. As controlled clinical trials investigating various sequences of ALK TKIs across multiple treatment lines will presumably never become available, real-world data can add important information to the body of evidence. For instance, a retrospective analysis conducted across 23 Italian centers showed that sequential administration of crizotinib and a next-generation ALK TKI provided remarkable clinical benefits in a real-life population of 290 patients [29].

Lorlatinib appears to be an ideal choice in the later-line setting, given its uniquely broad activity against *ALK* resistance mutations. According to in-vitro data, most of the commonly observed secondary *ALK* mutations are inhibited by lorlatinib [13]. The pivotal clinical trial demonstrated rapid, deep and durable responses with lorlatinib after up to three prior ALK TKIs that had been administered with or without chemotherapy [21,28]. Furthermore, it has shown an improvement in global quality of life for the majority of patients treated with lorlatinib [30]. Hypercholesterolemia and hypertriglyceridemia represented the most common treatment-related AEs, and the antitumor activity of lorlatinib was seen across a range of *ALK* resistance mutations, including G1202R.

In our study, we collected real-world data for lorlatinib therapy in heavily pretreated Caucasian patients with a long follow-up time. We achieved a maturity of our data of 79.3% and 67.6% for the duration of treatment and OS for *ALK*-positive patients, and 57.1% and 42.9% for *ROS1*-positive patients.

Our findings showed a relevant efficacy of lorlatinib for *ALK*-positive NSCLC even after multiple lines of treatment matching the results of the pivotal trial, with a median DoT of 4.4 months, median OS from lorlatinib start of 10.2 months, median OS from Stage III/IV of 41.8 months and an ORR of 43.2%.

We looked further into the patients who received only one second generation ALK TKI (n = 20) and compared their OS to those who had more prior lines of second generation TKIs (n = 17). Although there has been a numeric difference in the ORR, with 50% versus 35% in favour of patients who have received only one second generation TKI, our analysis indicated that they are significantly similar. Comparing patients with different amounts of prior TKI lines in general showed no difference between groups. Lorlatinib even demonstrated activity when administered after brigatinib, which is of particular interest, as data on this sequence have been lacking to date. In the phase II pivotal study, most of the 139 patients included had received alectinib as their last ALK TKI treatment prior to lorlatinib (n = 62), whereas brigatinib had been administered prior to lorlatinib in only eight of the patients [17]. In our data, we identified 19 patients who had brigatinib as their last line of therapy. That patients showed an ORR of 32.6% and DoT of 3.5 months. Although there was no statistically significant difference between the groups, it should be pointed out, that patients who had alectinib prior to lorlatinib (n = 15) showed higher response rates and DoT (ORR 60.0%; DoT 8.5 m). This observation was still made when analyzing patients with only one second generation TKI prior to lorlatinib (prior alectinib ORR 66.7% and DoT 10,0m vs. prior brigatinib ORR 36.4% and DoT 3.5 m). These findings should be interpreted with care and need to be investigated in following studies. In conclusion, there was no significant difference in the DoT, OS and ORR regarding the line or sequence and lorlatinib may be administered in earlier as well as later lines of therapy and also after second generation TKIs.

A recently published study providing real-life data for lorlatinib in a later line setting for mainly Asian patients showed comparable results for the extracranial response rate (22%, n = 59) and median DoT (5.6 months) in *ALK*-positive patients. Differences can, however, be found concerning survival rates from diagnosis of advanced disease, as we reported a 2-year OS of 70.3% (comparing 95.8%, n = 76) and 5-year OS of 33.3% (compared to 79.4%). This can perhaps be partly explained by the difference in ethnicity and less heavily pretreated population (≥3 prior systemic lines in 90% of our *ALK*-patients vs. 67%) [31].

Moreover, our findings indicate notable activity of lorlatinib in individuals with *ROS1* rearrangements. A higher proportion of these patients, relative to those with *ALK*-positive disease, were receiving ongoing treatment at the time of analysis, and their median OS was longer. However, the limited patient numbers preclude firm conclusions on this, and lorlatinib has not been approved for clinical use in patients with *ROS1*-positive disease, to date.

Lorlatinib treatment was well tolerated. The majority of events did not contribute significantly to the symptom burden, which is an important aspect in the later-line setting. Hypercholesterolemia and hypertriglyceridemia occurred as the most common events. Only one patient had to permanently discontinue treatment due to an AE. AE rates were lower than the rates reported in the clinical trial setting, which is due to the observational nature. Moreover, the retrospective nature of this analysis entails a series of general limitations.

Due to the lack of sufficient data to the exact time of radiologic disease progression, we have not been able to calculate the progression free survival (PFS). As a surrogate marker, we used DoT, including treatment beyond progression. As some of the patients had received lorlatinib in later lines, without many reasonable alternative treatment options, there might be a discrepancy between DoT and PFS.

The question of whether lorlatinib therapy prevented the development of brain metastases cannot be fully answered based on our observations here, because patients without known CNS lesions and without neurologic symptoms did not undergo brain imaging at baseline due to standard of care. Meaningful data for intracranial response (ORR 62.5%) was possible to collect only from a small number of patients who had confirmed brain metastases at initiation of lorlatinib and did not receive any cranial radiation.

In addition, we have not generated data on resistance mutations, as testing for these mutants is not routinely conducted, which is often due to the difficulty in obtaining tissue and the knowledge that next-generation ALK inhibitors appear to be active regardless of the presence of resistance mutations. Nevertheless, the jury is still out regarding biomarker-driven treatment decisions in *ALK*-positive lung cancer, and investigations in this area are ongoing. The ALK Master Protocol study is currently exploring the usefulness of mutation testing using liquid biopsies followed by mutation-specific ALK TKI treatments in patients with *ALK*-positive, stage IV NSCLC who have experienced progression on next-generation ALK inhibitors (NCT03737994).

Although the broad mutational coverage of lorlatinib predisposes it for later-line treatment, it is conceivable that this very feature provides pronounced clinical benefit in the first line setting. In fact, the pivotal trial that was conducted in both treatment-naïve and previously treated patients showed an ORR of 90% in the treatment-naive patient group [28]. In comparison, response rates were markedly lower in patients who received lorlatinib after one or more of the other ALK TKIs.

Overall, the findings presented here might contribute to filling a data gap with respect to later-line use of lorlatinib under real-world conditions. They suggest relevant clinical activity of this therapy even in massively pretreated patients, with good tolerability.

## 4. Materials and Methods

We conducted a retrospective, multicenter, real-world analysis on patients with *ALK-* or *ROS1*-positive NSCLC who had undergone treatment with lorlatinib as second or later line therapy. Prior to lorlatinib, all *ALK*-positive patients had received at least one second generation ALK TKI. *ROS1*-positive patients had received at least one first or second generation TKI. The standard starting dose of lorlatinib was 100 mg/day.

Patients included were treated in 5 lung cancer centers in Austria as part of the Pfizer global compassionate use program between January 2016 and May 2020. All data were extracted from medical records. The primary outcome duration of treatment (DoT) was defined as the time from initiation of lorlatinib to last administered dose. Overall survival (OS) was defined as the time from first lorlatinib dose to date of death. In addition, we calculated the overall survival (OS2) defined as time from diagnosis of locally advanced or metastatic disease to death.

The best overall response was assessed by the treating investigators determined by radiologic complete response (CR), partial response (PR), stable disease (SD) and progressive disease (PD). Overall response rate (ORR) was defined as the sum of CR and PR, and disease control rate (DCR) was defined as the sum of CR, PR and SD compared to the overall number of patients.

Adverse events (AEs) were graded as per Common Terminology Criteria for Adverse Events (CTCAE) version 5.0.

### Statistical Analysis

Median DoT and median OS as well as the number at risk were analyzed using the Kaplan–Meier-estimator and a confidence interval (CI) of 95%. Confidence interval for proportions like ORR was calculated using the Clopper–Pearson method.

The data cut-off was May 21, 2020. Patients were censored for the analysis if they were still alive for OS or on treatment for DoT. The median follow-up time was calculated from the time of first dose of lorlatinib to the data cut-off.

For a comparison of subgroups, the Log-rank test with a level of significance of 5% (chi square *p* = 0.05) was used. The first subgroup comparison in *ALK*-positive patients was done by separating the number of prior TKI lines, and the second subgroups are defined according to whether they received more than one second generation TKI. All statistical analysis where conducted using SPSS v.26.0 (IBM SPSS Statistics).

## 5. Conclusions

This retrospective analysis indicates that patients with advanced *ALK*- or *ROS1*-positive NSCLC who have been previously treated with ALK-TKIs can be treated in later lines with lorlatinib for prolonged periods of time. Lorlatinib was well tolerated and was also active for the *ROS1*-positive population. Further evidence is required to confirm these observations. For the time being, the question remains as to whether to use third generation ALK inhibitors as later lines, rather than in the early treatment setting.

## Figures and Tables

**Table 1:** Baseline Patient Characteristics.

Patient Characteristics	ALK(+)	ROS(+)
**N (%)**	37 (72.5%)	14 (27.5%)
**Age at metastatic diagnosis**		
Mean age (SD) at met. diagnosis	53.0 (13.4)	55.4 (16.4)
Range	29–77	26–82
**Sex**		
Female	24 (64.9%)	7 (50.0%)
Male	13 (35.1%)	7 (50.0%)
**Smoking history**		
Current	3 (8.1%)	1 (7.1%)
Former	11 (29.7%)	5 (35.7%)
Never-Smoker	23 (62.2%)	8 (57.1%)
**Stage at initial diagnosis**		
Stage I	1 (2.7%)	1 (7.1%)
Stage II	0 (0%)	0 (0%)
Stage IIIa	3 (8.1%)	0 (0%)
Stage IIIb	3 (8.1%)	0 (0%)
Stage IV	30 (81.1%)	13 (92.9%)
**Brain metastasis at diagnosis**		
Yes	19 (51.4%)	9 (64.3%)
No	15 (40.5%)	4 (28.6%)
Unknown	3 (8.1%)	1 (7.1%)
**Method of detection**		
IHC	13 (35.1%)	5 (35.7%)
FISH	17 (45.9%)	7 (50.0%)
NGS	1 (2.7%)	0 (0%)
More than 1	6 (16.2%)	2 (14.3%)
**Histology**		
Adenocarcinoma	35 (94.6%)	13 (92.8)
Adeno-squamous	1 (2.7%)	1 (7.1%)
Squamous-cell carcinoma	1 (2.7%)	0 (0%)
**Prior lines of therapy**		
2 lines	3 (8.1%)	6 (42.9%)
3 lines	11 (29.7%)	2 (14.3%)
4 lines	14 (37.8%)	1 (7.1%)
5 lines	8 (21.6%)	4 (28.6%)
6 lines	0 (0%)	1 (7.1%)
9 lines	1 (2.7%)	0 (0%)
**Prior lines of TKI**		
1 lines	10 (27.0%)	11 (78.6%)
2 lines	13 (35.1%)	3 (21.4%)
3 lines	13 (35.1%)	0 (0%)
4 lines	1 (2.7%)	0 (0%)
**Prior lines in detail**		
Alectinib	14 (37.8%)	0 (0%)
Brigatinib	27 (73.0%)	0 (0%)
Ceritinib	21 (56.8%)	3 (21.4%)
Crizotinib	25 (67.6%)	14 (100%)
At least one Chemotherapy	10 (27.0%)	8 (57.1%)

ALK: anaplastic lymphoma kinase; SD: standard deviation; IHC: immunohistochemistry; FISH: fluorescence in situ hybridization; NGS: next generation sequencing; TKI: tyrosine kinase inhibitor.

**Table 2:** Efficacy results of ALK and ROS positive patients including risk analysis

**Efficacy Results of ALK Positive Patients**						
**Results**	**Overall**	**1 Prior TKI**	**2 Prior TKI**	**≥3 Prior TKI**	**Only One 2nd Gen TKI**	**≥Two 2nd Gen TKI**
**N** (**%**)	37 (100%)	10 (27.0%)	13 (35.1%)	14 (37.8%)	20 (54.1%)	17 (45.9%)
**ORR** **^1^**	43.2% (27.1; 60.5)	40.0% (12.2; 73.8)	53.8% (25.1; 80.8)	35.7% (12.8; 64.9)	50.0% (27.2; 72.8)	35.3% (14.2; 61.7)
**DCR** **1**	56.8% (39.5; 72.9)	60.0% (26.2; 87.8)	69.2% (38.6; 90.9)	42.9% (17.7; 71.1)	60.0% (36.1; 80.9)	52.9% (27.8; 77.0)
**CR**	1 (2.7%)	0 (0%)	0 (0%)	1 (7.1%)	0 (0.0%)	1 (5.9%)
**PR**	15(40.5%)	4 (40.0%)	7 (53.8%)	4 (28.6%)	10 (50.0%)	5 (29.4%)
**SD**	5 (13.5%)	2 (20.0%)	2 (15.4%)	1 (7.1%)	3 (10.0%)	3 (17.6%)
**PD**	16 (43.2)	4 (40.0%)	4 (30.8%)	8 (57.1%)	8 (40.0%)	8 (47.1%)
**Median DoT** **^2^**	4.4 (1.3; 7.6)	4.4 (0.5; 8.2)	NR	3.0 (1.8; 4.2)	4.4 (1.1; 7.7)	4.4 (0.2; 8.6)
**Median OS2** (**since lorla start**)	10.2 (3.6; 16.8)	6.4 (3.9; 9.0)	31.2 (NR)	7.1 (0.0; 20.2)	7.9 (0.0; 29.7)	10.2 (4.1; 16.2)
**Median OS2** (**since advanced diagnosis**)	41.8 (34.1; 49.5)	28.3 (11.9; 44.8)	66.5 (NR)	40.6 (30.5–50.8)	39.2 (21.7; 56.7)	43.4 (30.9; 55.9)
**Risk analysis of the efficacy data of ALK positive patients**						
**Risk Analysis** **^3^**	**Maturity**	**3-Months**	**6-Months**	**12-Months**	**2-Years**	**5-Years**
**DoT rate**	70.3%	62.2%	45.9%	31.7%	NR	NR
**OS rate** (**since lorla start**)	67.6%	73.0%	62.2%	45.4%	35.1%	NR
**OS rate** (**since advanced diagnosis**)	67.6%	100%	100%	86.5%	70.3%	33.3%
**Efficacy results of ROS1 positive patients**						
**Results**	**Overall**		**1 Prior TKI**		**2 Prior TKI**	
**N** (**%**)	14 (100%)		11 (78.6%)		3 (21.4%)	
**ORR ^4^**	85.7% (57.2; 98.2)		90.9% (58.7; 99.8)		66.7% (0.9; 99.2)	
**DCR ^1^**	92.9% (66.1; 99.8)		100% (71.5; 100)		69.2% (0.9; 99.2)	
**CR**	2		2		0	
**PR**	10		8		2	
**SD**	1		1		0	
**PD**	1		0		1	
**Median DoT ^5^**	12.2 (0.0; 29.5)		25.0 (8.0; 42.1)		7.0 (0.0; 16.6)	
**Median OS ^5^** (**since lorla start**)	20.0 (NR)		17.6 (NR)		24.4 (3.1; 45.6)	
**Median OS ^5^** (**since advanced diagnosis**)	40.1 (NR)		39.2 (NR)		46.3 (38.7; 105.9)	
**Risk analysis of the efficacy data of ROS1 positive patients**						
**Risk Analysis ^6^**	**Maturity**	**3-Months**	**6-Months**	**12-Months**	**2-Years**	**5-Years**
**DoT rate** (**SD**)	57.1%	78.6%	78.6%	50.0%	49.0%	NR
**OS rate** (**SD**) (**since lorla start**)	42.9%	92.9%	92.9%	61.4%	50.8%	NR
**OS rate** (**SD**) (**since advanced diagnosis**)	42.9%	100%	100%	100%	92.9%	58.8%

^1^ 95% confidence interval was calculated using the Clopper–Pearson method;^2^ the median was calculated with the Kaplan–Meier-estimator; ^3^ the risk analysis was an estimation of the cumulative incidence for the event of interest calculated using the Kaplan–Meier method; ^4^ the 95% confidence interval was calculated using the Clopper–Pearson method; ^5^ the median was calculated with the Kaplan–Meier-estimator; ^6^ the risk analysis was an estimation of the cumulative incidence for the event of interest calculated using the Kaplan–Meier method; ALK: *anaplastic lymphoma kinase; TKI: tyrosine kinase inhibitor; ORR: overall response rate; DCR: disease control rate; CR: complete response; PR: partial response; SD: stable disease; PD: progressive disease; DoT: duration on treatment; NR: not reached; OS: overall survival*

**Table 3:** Adverse Events.

Adverse Events	Grade 1	Grade 2	Grade 3	Grade 4
**N = 51**	**N (%)**	**N (%)**	**N (%)**	**N (%)**
Hyperlipidemia	8 (16%)	8 (16%)	4 (8%)	5 (10%)
Hypercholesterolemia	6 (12%)	4 (8%)	2 (4%)	2 (4%)
Hypertriglyceridemia	2 (4%)	4 (8%)	2 (4%)	3 (6%)
Peripheral Edema	5 (10%)	1 (2%)	1 (2%)	
Cognitive Effects	1 (2%)	2 (4%)		
Diarrhea	1 (2%)	2 (4%)		
Arthralgia				1 (2%)
Fatigue				1 (2%)
Peripheral Neuropathy	1 (2%)			
Other	3 (6%)		2 (4%)	
Bloating	1 (2%)			
Muscle Weakness	1 (2%)			
Myalgia	1 (2%)			
Pneumonitis			1 (2%)	
Thrombosis			1 (2%)	
